# Supplementary material for: In-Depth Chemical Characterization of Punica granatum L. Seed Oil
Source: Foods. 2023 Apr 9;12(8):1592. doi: 10.3390/foods12081592 (PMC10138243; doi:10.3390/foods12081592)
Supplement: Supplementary file 1 [file foods-12-01592-s001.zip › foods-2290307-supplementary.pdf]

Table S1. Retention time, calibration curve and correlation coefficient of reference compounds.

	Retention time	Calibration curve (µg/mL)	Correlation coefficient
Gallic acid	4.86	y = 15.51x + 37.06	0.9987
Catechin	15.48	y = 5.18 x - 24.29	0.9961
Chlorogenic acid	18.01	y =12.02x - 3.95	0.9991
Caffeic acid	19.01	y = 35.23x – 28.86	0.9989
Epicatechin	20.98	y = 2.47x + 58.32	0.9982
p-Coumaric acid	24.81	y = 42.12x – 19.25	0.9987
Cyanidin-3-rut	27.01	y = 16.58x + 34.53	0.9987
Ferulic acid	27.38	y = 20.65x + 22.96	0.9993
Rutin	27.75	y= 13.75x – 7.57	0.9979
Sinapic acid	27.91	y = 11.37x + 9.92	0.9987
Quercetin-3 gal	29.75	y= 49.69x + 34.98	0.9999
Myricetin	32.40	y = 21,51x – 5.93	0.9991
Quercetin	37.78	y = 21.69x + 24.12	0.9995
Kaempferol	42.25	y = 25.94x + 27.50	0.9988
Carvacrol	52.81	y= 4.03x + 22.29	0.9997
Timol	55.65	y= 4.93x + 33.21	0.9998

Table S2. Calculated monoisotopic masses and molecular weights of possible TAG species. All masses reported are for sodium adduct ions. The sequence of the fatty acid residues does not reflect their actual positions on the glycerol backbone, but they are by convention ordered from lower to higher mass. Palmitic acid – (16:0); linolenic acid – (18:3); linoleic acid – (18:2); oleic acid – (18:1); stearic acid – (18:0).

**Monoisotopic mass**	**Molecular weight**	**TAG**
829.726	830.31	(16:0); (16:0); (16:0)
851.710	852.32	(16:0); (16:0); (18:3)
853.726	854.33	(16:0); (16:0); (18:2)
855.742	856.35	(16:0); (16:0); (18:1)
857.757	858.37	(16:0); (16:0); (18:0)
873.695	874.32	(16:0); (18:3); (18:3)
875.710	876.34	(16:0); (18:3); (18:2)
877.726	878.35	(16:0); (18:2); (18:2)
877.726	878.35	(16:0); (18:3); (18:1)
879.742	880.37	(16:0); (18:3); (18:0)
879.742	880.37	(16:0); (18:2); (18:1)
881.757	882.39	(16:0); (18:1); (18:1)
881.757	882.39	(16:0); (18:2); (18:0)
883.773	884.40	(16:0); (18:1); (18:0)
885.789	886.42	(16:0); (18:0); (18:0)
895.679	896.33	(18:3); (18:3); (18:3)
897.695	898.34	(18:3); (18:3); (18:2)
899.710	900.36	(18:3); (18:2); (18:2)
899.710	900.36	(18:3); (18:3); (18:1)
901.726	902.38	(18:2); (18:2); (18:2)
901.726	902.38	(18:3); (18:3); (18:0)
901.726	902.38	(18:3); (18:2); (18:1)
903.742	904.39	(18:3); (18:1); (18:1)

903.742	904.39	(18:2); (18:2); (18:1)
903.742	904.39	(18:3); (18:2); (18:0)
905.757	906.41	(18:2); (18:1); (18:1)
905.757	906.41	(18:2); (18:2); (18:0)
905.757	906.41	(18:3); (18:1); (18:0)
907.773	908.42	(18:3); (18:0); (18:0)
907.773	908.42	(18:1); (18:1); (18:1)
907.773	908.42	(18:2); (18:1); (18:0)
909.789	910.44	(18:2); (18:0); (18:0)
909.789	910.44	(18:1); (18:1); (18:0)
911.804	912.46	(18:1); (18:0); (18:0)
913.820	914.47	(18:0); (18:0); (18:0)

Table S3. Theoretical isotopic distribution of TAGs displayed in Figure 1. (P: palmitic acid – (16:0); Ln: linolenic acid – (18:3); L: linoleic acid – (18:2); O: oleic acid – (18:1); S: stearic acid – (18:0)

**(16:0); (16:0); (18:3) = PPLn**
851.710, 100.00
852.714, 58.66
853.717, 18.12
854.720, 3.91
855.723, 0.66
856.726, 0.091
857.729, 0.011

**(18:3); (18:3); (18:3) = LnLnLn**
895.679, 100.00
896.682, 62.94
897.686, 20.70
898.689, 4.72
899.692, 0.84
900.695, 0.12
901.698, 0.02

**(16:0); (18:3); (18:3) = PLnLn**
873.695, 100.00
874.698, 60.80
875.701, 19.39
876.705, 4.30
877.708, 0.74
878.710, 0.11
879.714, 0.01

**(18:3); (18:3); (18:2) = LnLnL**
897.695, 100.00
898.698, 62.96
899.701, 20.72
900.705, 4.73
901.708, 0.84
902.711, 0.12
903.714, 0.02

**(16:0); (18:3); (18:2) = PLnL**
875.710, 100.00
876.714, 60.82
877.717, 19.41
878.720, 4.31
879.723, 0.74
880.726, 0.11
881.729, 0.01

**(18:3); (18:3); (18:1) = LnLnO**
899.710, 100.00
900.714, 62.98
901.717, 20.73
902.720, 4.73
903.723, 0.84
904.726, 0.12
905.729, 0.02

**(16:0); (18:3); (18:2) = PLnO**
877.726, 100.00
878.730, 60.84
879.733, 19.42
880.736, 4.32
881.739, 0.74
882.742, 0.10
883.748, 0.013

**(18:3); (18:3); (18:0) = LnLnS**
901.726, 100.00
902.730, 63.01
903.733, 20.75
904.736, 4.74
905.739, 0.84
906.742, 0.12
907.745, 0.02

**(18:1); (18:0); (18:0) = OSS**
911.80436, 100.00
912.80778, 63.12
913.81104, 20.82
914.81419, 4.76
915.81725, 0.84
916.82026, 0.12
917.82321, 0.02

**(18:0); (18:0); (18:0) = SSS**
913.82001, 100.00
914.82343, 63.14
915.82669, 20.83
916.82984, 4.77
917.83291, 0.85
918.83591, 0.12
919.83887, 0.02

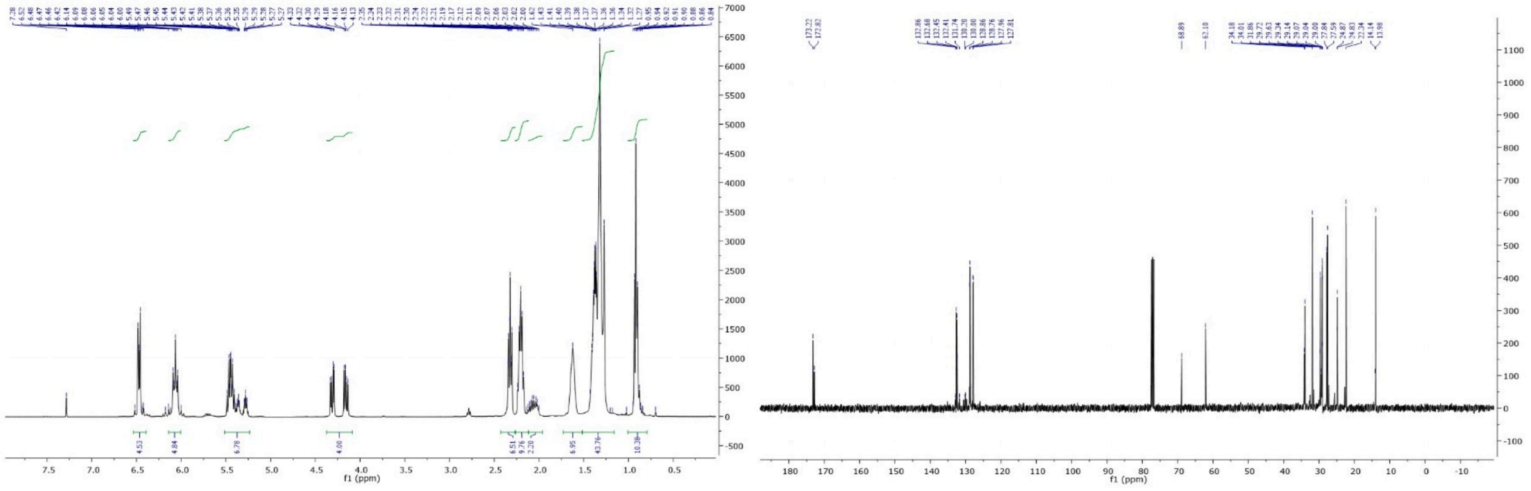

Figure S1. ^{1}H and ^{13}C NMR of Soxhlet oil, **1**.

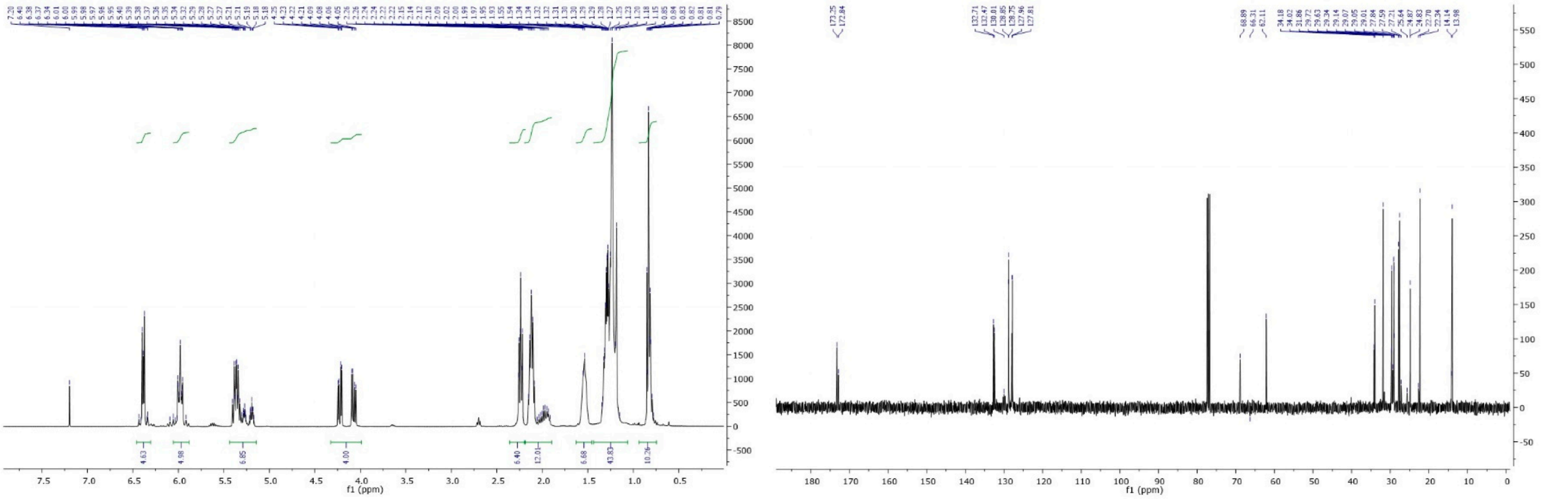

Figure S2. ^{1}H and ^{13}C NMR of $scCO_2$ oil, **2**.

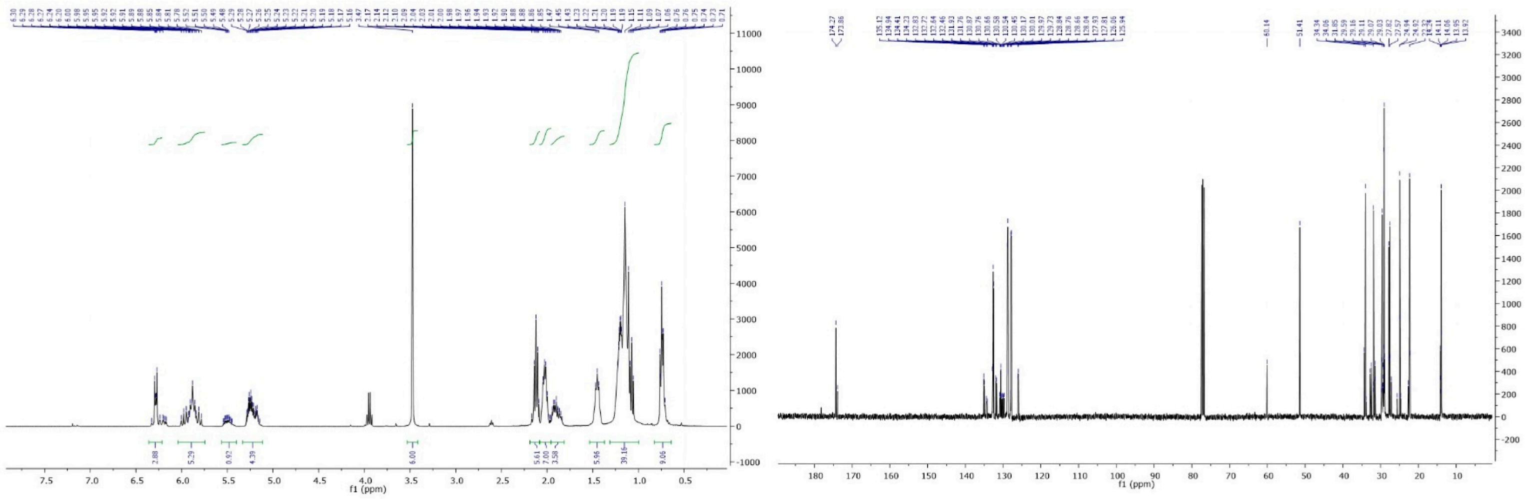

Figure S3. ^{1}H and ^{13}C NMR inverse gated of transesterified Soxhlet oil, **1a**.

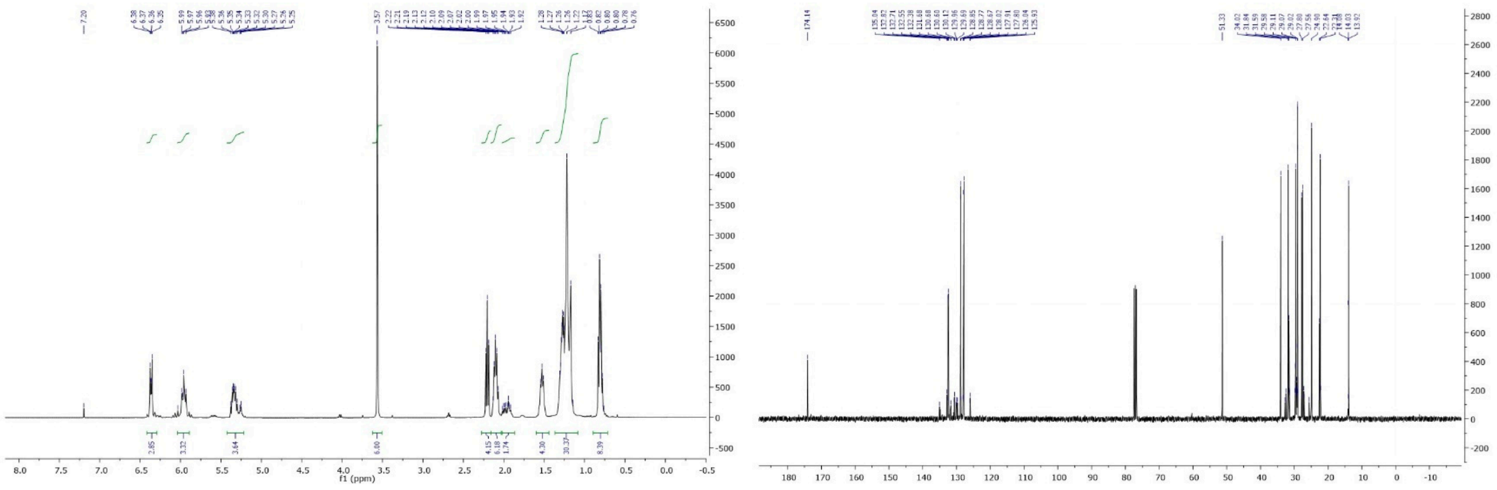

Figure S4. ^{1}H and ^{13}C NMR inverse gated of transesterified $scCO_2$ oil **2a**.

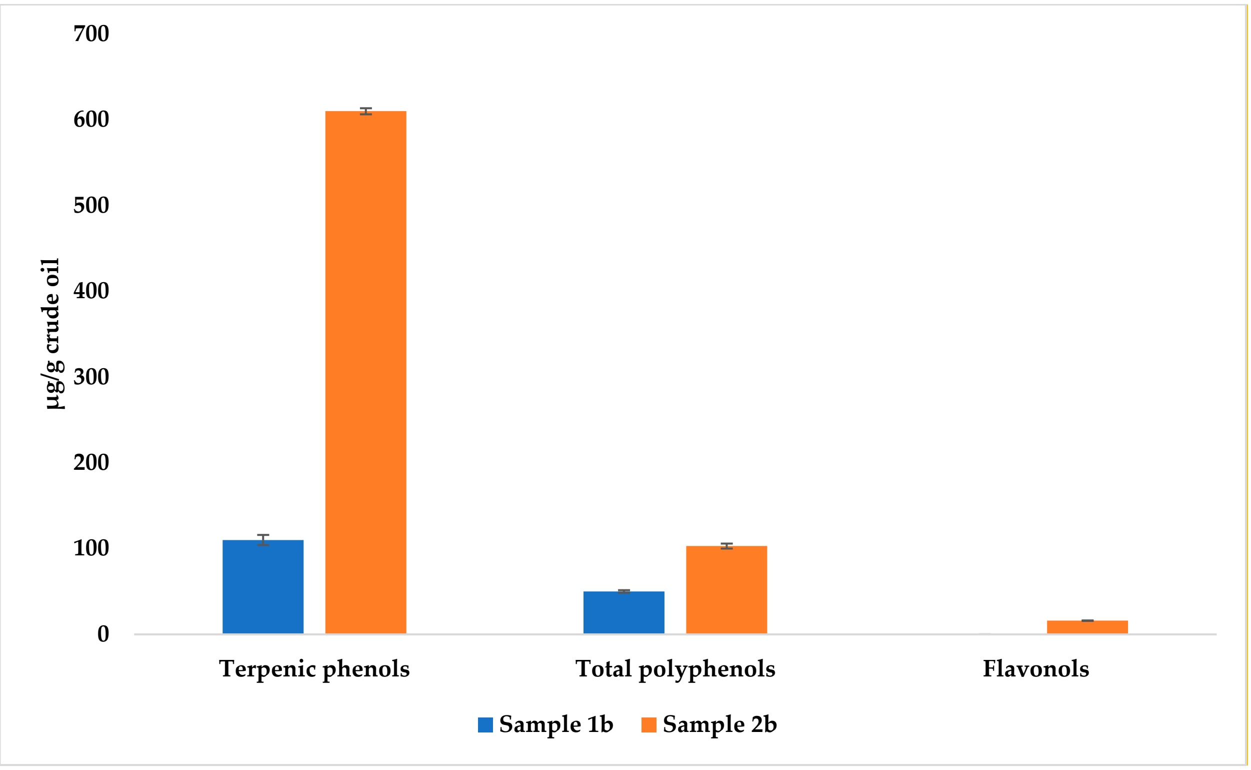

Figure S5. Differences in the polyphenolic content of SPE Soxhlet oil**, 1b** and scCO_2 oil**, 2b.**
